# Modelling the impact of chlamydia screening on the transmission of HIV among men who have sex with men

**DOI:** 10.1186/1471-2334-13-436

**Published:** 2013-09-18

**Authors:** Maria Xiridou, Henrike J Vriend, Anna K Lugner, Jacco Wallinga, Johannes S Fennema, Jan M Prins, Suzanne E Geerlings, Bart JA Rijnders, Maria Prins, Henry JC de Vries, Maarten J Postma, Maaike G van Veen, Maarten F Schim van der Loeff, Marianne AB van der Sande

**Affiliations:** 1 National Institute of Public Health and Environment, P.O. Box 1, 3720, BA Bilthoven, the Netherlands; 2 Department of Internal Medicine, Academic Medical Centre, Amsterdam, the Netherlands; 3 STI Outpatient Clinic, Public Health Service of Amsterdam, Amsterdam, the Netherlands; 4 Department of Internal Medicine and Infectious Diseases, Erasmus University Medical Centre, Rotterdam, the Netherlands; 5 Research Department, Public Health Service of Amsterdam, Amsterdam, the Netherlands; 6 Department of Dermatology, Academic Medical Centre, Amsterdam, the Netherlands; 7 Department of Pharmacy, University of Groningen, Groningen, the Netherlands; 8 Julius Center, University Medical Centre, Utrecht, the Netherlands

**Keywords:** HIV, Chlamydia trachomatis, Men who have sex with men, Mathematical model, Chlamydia screening

## Abstract

**Background:**

Recent studies have found high prevalences of asymptomatic rectal chlamydia among HIV-infected men who have sex with men (MSM). Chlamydia could increase the infectivity of HIV and the susceptibility to HIV infection. We investigate the role of chlamydia in the spread of HIV among MSM and the possible impact of routine chlamydia screening among HIV-infected MSM at HIV treatment centres on the incidence of chlamydia and HIV in the overall MSM population.

**Methods:**

A mathematical model was developed to describe the transmission of HIV and chlamydia among MSM. Parameters relating to sexual behaviour were estimated from data from the Amsterdam Cohort Study among MSM. Uncertainty analysis was carried out for model parameters without confident estimates. The effects of different screening strategies for chlamydia were investigated.

**Results:**

Among all new HIV infections in MSM, 15% can be attributed to chlamydia infection. Introduction of routine chlamydia screening every six months among HIV-infected MSM during regular HIV consultations can reduce the incidence of both infections among MSM: after 10 years, the relative percentage reduction in chlamydia incidence would be 15% and in HIV incidence 4%, compared to the current situation. Chlamydia screening is more effective in reducing HIV incidence with more frequent screening and with higher participation of the most risky MSM in the screening program.

**Conclusions:**

Chlamydia infection could contribute to the transmission of HIV among MSM. Preventive measures reducing chlamydia prevalence, such as routine chlamydia screening of HIV-infected MSM, can result in a decline in the incidence of chlamydia and HIV.

## Background

In several countries, high prevalences of asymptomatic rectal chlamydia have been found among HIV-infected men who have sex with men (MSM) [1-4]. Most of these infections remain undetected and untreated. Therefore, screening for chlamydia among HIV-infected MSM may considerably reduce the prevalence of chlamydia in this specific subpopulation. In addition, certain studies suggest that chlamydia screening among MSM may result in a decline in HIV transmission, because infection with chlamydia may increase the transmissibility of HIV (for HIV-infected individuals) and the susceptibility to HIV infection (for HIV-negative individuals) [5-11]. However, the benefits of chlamydia screening are still unclear; for MSM, this is because of the perceived mildness of complications of chlamydia infection in men.

In this study, we investigated the role of rectal chlamydia in the spread of HIV among MSM, using a dynamic transmission model. An increase in HIV infectivity and susceptibility in individuals with chlamydia was included in the model. Also, we accounted for the impact of antiretroviral therapy (ART) in reducing HIV infectivity and in eliminating the increase in HIV infectivity due to co-infection with chlamydia. We examined how chlamydia affects the spread of HIV in the population, by calculating the fraction of new HIV infections attributable to chlamydia infection. Subsequently, we studied the impact of chlamydia screening among HIV-infected MSM at HIV treatment centres on the incidence of chlamydia and HIV in the MSM population. To reveal the dependence of the results on the assumption of increased HIV infectivity and susceptibility due to chlamydia, the results were calculated with different levels of this increase.

## Methods

### The model

We developed a deterministic compartmental model that describes the transmission of HIV and chlamydia among sexually active MSM. In the model, infection with HIV or with chlamydia occurs only via unprotected anal intercourse (UAI) between men. Three types of partnerships are distinguished in the model: steady partners, single-act casual partners (with whom they have only one sexual contact and that is UAI), and multiple-acts casual partners (with whom they have multiple sexual contacts of which at least one is UAI). The population is stratified into four sexual risk groups, with increasing level of sexual risk behaviour: low, fairly high, very high, and extremely high (the last three are referred to as high risk groups). The level of risk behaviour is determined by the total number of sexual partners with whom men have UAI. Low risk MSM have no UAI with casual partners. High risk MSM have UAI with casual partners; the total number of partners is highest in the extremely high risk group and lowest in the fairly high risk group (Additional file 1: Table S2).

**Figure 1:**
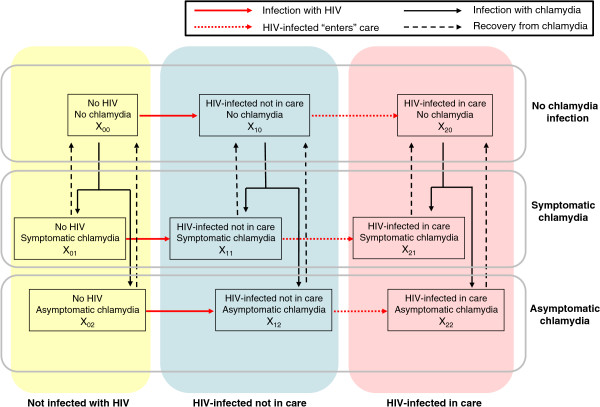
**Flow diagram of the model describing transmission of HIV and chlamydia.** The population consists only of men who have sex with men (MSM). The solid black arrows show infection with chlamydia and the solid red arrows show infection with HIV. HIV-infected MSM not in care may “enter care” and move to the class of HIV-infected MSM in care (dashed red arrows) after a positive HIV-test and registration at one of the specialised HIV treatment centres. Recovery from chlamydia infection (with or without treatment) is shown with dashed black arrows. For the variables *X*_*ij*_ the first subscript denotes status of HIV infection (0, not infected with HIV; 1, HIV-infected not in care; 2, HIV-infected in care), while the second subscript denotes status of chlamydia infection (0, no chlamydia infection; 1, symptomatic chlamydia; 2, asymptomatic chlamydia). The yellow shaded area denotes MSM not infected with HIV, the blue shaded area denotes HIV-infected MSM not in care, and the pink shaded area denotes HIV-infected MSM in care.

The population is also stratified according to state of HIV infection and state of chlamydia infection. Three states of HIV infection are distinguished in the model: not infected with HIV, HIV-infected not in care, and HIV-infected in care (Figure 1). HIV-infected MSM not in care are unaware of their infection or they are aware of their infection but have not (yet) been registered at a specialised HIV treatment centre. HIV-infected MSM in care are aware of their infection and have been registered at a specialised HIV treatment centre; they receive regular clinical care and initiate antiretroviral therapy (ART) guided by their CD4 counts. Most of the HIV-infected men in care receive ART [12]; hence, in the model, HIV infectivity for those in care is lower than HIV infectivity for those not in care, due to ART. For chlamydia infection, three states are distinguished: susceptible to chlamydia, symptomatic chlamydia, and asymptomatic chlamydia (Figure 1). The duration of symptomatic chlamydia is generally much shorter than that for asymptomatic chlamydia due to care seeking behaviour and treatment. Those without symptoms usually remain undetected until natural recovery; with screening, they may be detected and treated and become susceptible (see details below for chlamydia screening).

The model is defined by a system of differential equations and the parameters are defined in Additional file 1: Tables S1–S3. The model was parameterized and calibrated to reflect the current situation among MSM in the Netherlands (see section about the uncertainty analysis).

### Increased HIV infectivity and susceptibility due to chlamydia infection

We assumed that for individuals infected with both HIV and chlamydia, the level of HIV infectivity is increased by a factor *v* (compared to HIV infectivity for individuals without chlamydia), due to co-infection with chlamydia [7-9]. For those infected with chlamydia but not HIV, the susceptibility to HIV infection is increased by a factor *φ* (compared to HIV susceptibility for those without chlamydia), due to chlamydia infection [10,11]. The number of new HIV infections that can be attributed to infection with chlamydia was calculated as the difference between the total number of new HIV infections and the number of new HIV infections if the two factors were both equal to one. Dividing this number by the total number of new HIV infections resulted in the fraction of new HIV infections attributable to infection with chlamydia.

### Uncertainty analysis and model calibration

To account for uncertainty in model parameters, a range of possible values was assigned to each uncertain parameter. Using Latin Hypercube Sampling [13], 10,000 sets of values were sampled from the uniform distribution on these ranges. The model equations were then solved numerically with each of the 10,000 parameter sets until the system reached a stable state; this is assumed to represent the current situation in the Netherlands (results with the current opportunistic screening rates for chlamydia – see details on screening scenarios below).

Then we calibrated the model to the current number of HIV-infected MSM in care at HIV treatment centres in the Netherlands (N = 8,523 in 2011 [14]). Let *Z*_*i*_ denote the number of HIV-infected MSM in care calculated from the model with the *i*-th set of parameter values, for *i* = 1,2,…,10,000. We calculated the Poisson likelihood of each estimate *Z*_*i*_ by assuming that the number of HIV-infected MSM in care follows the Poisson distribution with mean *Z*_*i*_. This likelihood represents the probability of observing the value 8,523, if the true expectation were *Z*_*i*_. In this way, each of the 10,000 estimates *Z*_*i*_ is assigned a likelihood representing how likely this estimate is, given our current knowledge. Subsequently, we selected the parameter sets with high likelihood: those with likelihood higher than 1/8 of the saturated likelihood (which is the likelihood of the value 8,523 from the Poisson distribution with mean 8,523): 0.0043212/8 = 0.00054. In this way, we selected the parameter combinations resulting in likely estimates of the number of HIV-infected MSM in care and excluded those resulting in unlikely estimates. In the remaining of the manuscript, we present results only from the selected parameter sets.

Next, the screening rates were modified to reflect changes in the testing frequency of MSM. The model equations were solved with the new screening rates and the selected parameter sets for the subsequent thirty years. For each outcome of interest (for example, prevalence of HIV) we report the median and the interquartile range (IQR: from the 25^th^ to the 75^th^ value) of the values calculated only with the selected parameters.

### Scenarios for chlamydia screening

#### Current opportunistic chlamydia screening

Currently in the Netherlands, there is no routine screening for chlamydia, but only opportunistic screening: individuals without chlamydia symptoms may be tested for chlamydia and other sexually transmitted infections (STIs) at their own initiative, at STI clinics or general practitioners. The current frequency of opportunistic screening was estimated as follows: low risk men are tested every 2.5-3.5 years; high risk men in care every 1–1.5 years; high risk men not infected with HIV or HIV-infected not in care every 1.5-2.5 years [1,15] (Additional file 1: Table S3).

#### Reduction in the frequency of the current opportunistic screening among MSM

To reveal the impact of the current opportunistic chlamydia screening on the incidence of chlamydia and HIV, we examined first a hypothetical scenario where the frequency of opportunistic screening is reduced. This could happen, for instance, if campaigns promoting chlamydia screening are relaxed or if the need to treat asymptomatic chlamydia is perceived as less important than it is now. This scenario was implemented by increasing the time interval between chlamydia tests by 50%: low risk men are tested every 3.75-5.25 years; high risk men in care every 1.5-2.25 years; high risk men who are not infected with HIV or HIV-infected not in care every 2.25-3.75 years.

#### Introduction of routine chlamydia screening of HIV-infected MSM in care at HIV treatment centres

Chlamydia screening among HIV-infected MSM in care could have an impact on reducing HIV transmission, because chlamydia infection may increase HIV infectivity. Moreover, chlamydia screening could be implemented during the existing visits of HIV-infected men at HIV treatment centres. To investigate the impact of such a program, we examined three hypothetical scenarios with different screening frequencies: every twelve months, every six months, or every four months. In these scenarios, it is assumed that: only HIV-infected MSM in care are screened; routine screening is added to (and not replacing) the current opportunistic screening; participation in the routine screening program is 100%, which means that all HIV-infected MSM in care are screened (and treated if positive) every twelve, six, or four months. The three scenarios are referred to as routine screening at HIV treatment centres; we present its impact on reducing HIV incidence in the whole MSM population.

#### Suboptimal participation in routine chlamydia screening of HIV-infected MSM in care at HIV treatment centres

Although HIV-infected MSM in care have regular consultations at HIV treatment centres, some of them may not participate in the routine screening program (for instance, because they have been recently tested at STI clinics, due to symptoms or known risk of exposure) or they may participate but not return to receive their medication, if they are found positive (mostly because of the lack of symptoms or hindrance [16]). In addition, recent data suggest that the current treatment may not be 100% effective [17]. To model these conditions, we recalculated the impact of one of the scenarios for routine screening assuming that participation is suboptimal (less than 100%); this was done for the scenario with screening every six months, also referred to as semi-annual screening. In order to examine the role of the different sexual risk groups, we studied four scenarios: in each scenario, participation was 80% in one of the four sexual risk groups and 100% in the other three risk groups:

80% participation of low risk HIV-infected MSM in care;

80% participation of fairly high risk HIV-infected MSM in care;

80% participation of very high risk HIV-infected MSM in care;

80% participation of extremely high risk HIV-infected MSM in care.

### Percentage change in incidence

For the above mentioned screening scenarios, we show the percentage change in HIV incidence and the percentage change in chlamydia incidence, calculated as

$$100\frac{\left(\mathrm{current}\;-\;\mathrm{new}\right)}{\mathrm{current}}$$

where, “current” is the incidence with the current opportunistic screening and “new” is the incidence with the new screening scenario.

## Results

### The interaction between HIV and chlamydia

First, we calculated from the model the prevalence and the incidence of HIV and of chlamydia infection, for the current situation in the Netherlands (with only opportunistic screening). The calculated median prevalence of HIV is 4.26% (IQR, 4.19-4.33%) and the median prevalence of chlamydia 2.69% (IQR, 1.68-3.71%) in the MSM population. However, large variations are observed in the prevalences of HIV and chlamydia within the population. Specifically, the prevalence of HIV varies from 0.5% in the lowest risk group to 72.8% in the highest risk group; the prevalence of chlamydia infections varies from 0.1% in the lowest risk group to 57.6% in the highest risk group (Additional file 1: Figure S1).

The median prevalence of chlamydia is 1.8% among MSM not infected with HIV, but 22.7% among HIV-infected MSM (Additional file 1: Figure S2). The high prevalence of chlamydia among HIV-infected MSM is mostly attributable to the high risk behaviour in this specific subgroup. From the model, it was calculated that MSM who have UAI with casual partners (high risk MSM) comprise 90.8% of the HIV-infected MSM population, but only 24.5% of the MSM not infected with HIV. This implies that the men who get infected with HIV are mostly those with high sexual risk behaviour (having UAI with casual partners) and because of their high risk behaviour they are also more likely to get infected with chlamydia.

**Figure 2:**
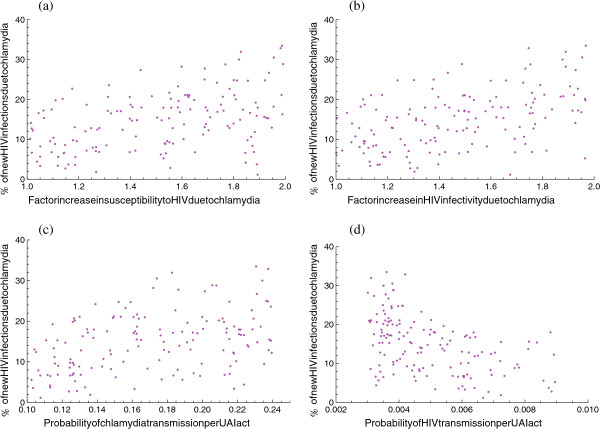
**The percentage of new HIV infections that can be attributed to chlamydia.** The percentage is plotted against four of the uncertain parameters: **(a)** the factor increase in susceptibility to HIV due to chlamydia; **(b)** the factor increase in HIV infectivity due to co-infection with chlamydia; **(c)** the probability of chlamydia transmission per act of unprotected anal intercourse (UAI); **(d)** the probability of HIV transmission per UAI act. In each plot, one point corresponds to one of the selected parameter sets: the value of the uncertain parameter in this set can be viewed on the horizontal axis, while the percentage of new HIV infections attributed to chlamydia (as calculated from the model with the specific parameter value) is shown on the vertical axis.

Among all new HIV infections, 15.2% (IQR, 8.9-19.8%) can be attributed to infection with chlamydia, specifically to the increased HIV infectivity and increased HIV susceptibility in those infected with chlamydia. This percentage is higher if the factors increasing HIV infectivity and HIV susceptibility due to chlamydia are higher, since, per definition, these factors determine the extra HIV infections attributable to chlamydia (Figure 2a,b). The percentage of HIV infections attributable to infection with chlamydia is also higher if chlamydia infectivity is higher, because chlamydia incidence is then higher and that intensifies the interaction with HIV; however, the percentage is lower if HIV infectivity is higher, since then the dynamics of HIV transmission are quite strong and can hardly be affected by chlamydia (Figure 2c,d).

### The impact of chlamydia screening on the incidence of HIV and of chlamydia

**Figure 3:**
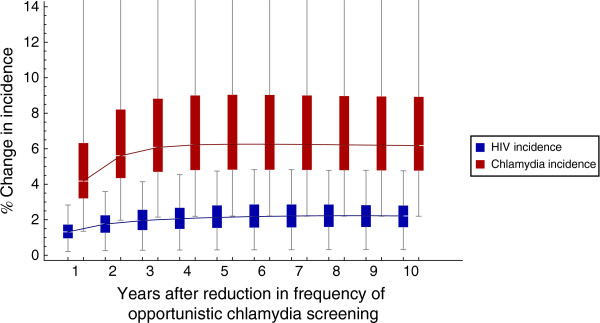
**Change in HIV and chlamydia incidence, after a reduction in the frequency of chlamydia screening.** Percentage change in the incidence of HIV (blue) and chlamydia (red) among MSM, after a reduction in the frequency of opportunistic chlamydia screening (the time interval between chlamydia tests as currently reported by MSM is increased by 50%). The percentage change is calculated compared to the current situation, with only opportunistic chlamydia screening (see Methods and Additional file 1: Table S3). In each year, the lines represent the medians; the blue and red boxes represent the interquartile range; the line segments above and below the boxes show the whole range of the results.

If MSM are less frequently tested for chlamydia, the incidence of both chlamydia and HIV may increase. In particular, increasing the screening period for chlamydia by 50% may lead to an increase of 6% in chlamydia incidence and 2% in HIV incidence among MSM (Figure 3 and Additional file 1: Figures S3-S4).

**Figure 4:**
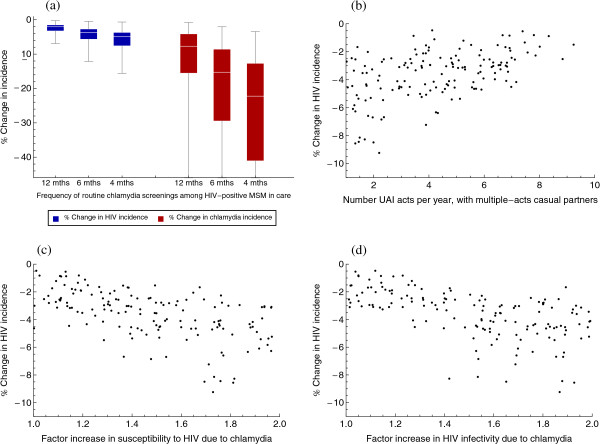
**Change in HIV and chlamydia incidence, due to routine chlamydia screening among HIV-infected MSM in-care.** Percentage change in the incidence of HIV or chlamydia among MSM, ten years after the introduction of routine chlamydia screening among HIV-infected MSM in care. The percentage change is calculated compared to the current situation, with only opportunistic chlamydia screening. **(a)** Percentage change in HIV (blue) and chlamydia (red) incidence, with routine chlamydia screening carried out every twelve, six, or four months. The boxes represent the interquartile range of the results; the white segment within the box represents the median of the results; the line segments above and below the boxes show the whole range of the results. **(b-d)** The percentage change in HIV incidence due to routine chlamydia screening of HIV-infected MSM in care every six months, plotted against three of the uncertain parameters: the number of acts of unprotected anal intercourse (UAI) per year per multiple-acts casual partner; the factor increase in susceptibility to HIV due to chlamydia; the factor increase in HIV infectivity due to co-infection with chlamydia.

The introduction of routine chlamydia screening of HIV-infected MSM in care can result in considerable reductions in the incidence of chlamydia, as well as of HIV (Figure 4). Ten years after the introduction of routine screening, the incidence of HIV among MSM is reduced by 2%, 4%, or 5% if routine screening is carried out every twelve, six, or four months, respectively; chlamydia incidence is reduced by 7%, 15%, or 22% if routine screening is carried out every twelve, six, or four months, respectively (Figure 4a). Chlamydia screening is more effective in reducing HIV incidence when the frequency of UAI with multiple-acts casual partners is low (Figure 4b and Additional file 1: Figure S5). This can be explained by the fact that with low UAI frequency the dynamics of HIV transmission are rather weak and that makes the impact of screening more prominent, while, with high UAI frequency, the dynamics of HIV transmission are too strong and can hardly be affected by chlamydia screening. Chlamydia screening is more effective in reducing HIV incidence also when the factors enhancing HIV infectivity and HIV susceptibility are high (Figure 4c,d and Additional file 1: Figure S5), because then the contribution of chlamydia to HIV transmission is higher, as shown in the previous paragraph.

**Figure 5:**
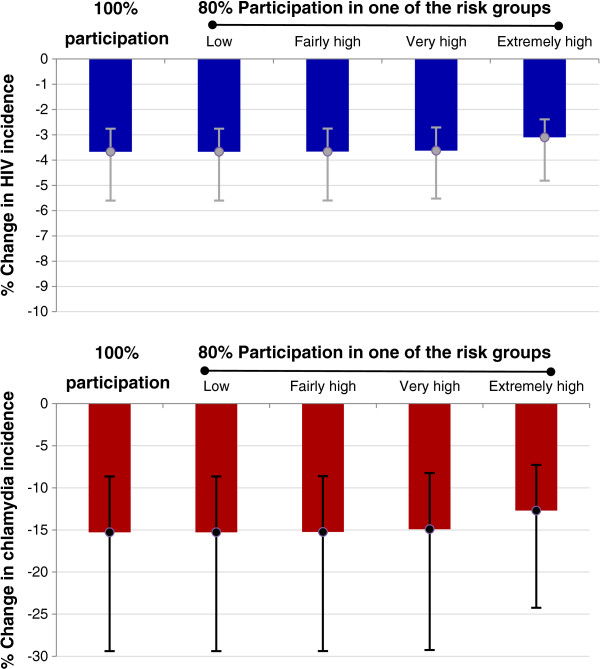
**The impact of suboptimal participation in routine chlamydia screening.** The percentage change in HIV incidence (blue) and chlamydia incidence (red), ten years after the introduction of routine chlamydia screening among HIV-infected MSM in care with suboptimal participation in the screening program: participation is 80% in only one of the four sexual risk groups and 100% in the other three risk groups. The four risk groups are: low risk, fairly high risk, very high risk, and extremely high risk, with increasing number of sexual partners. The results for the case with 100% participation of all HIV-infected MSM in care (first columns in both plots) are shown for comparison. The height of the columns shows the median and the line segments show the interquartile range of the results with the selected parameter sets.

The impact of screening as shown in Figure 4 will however be lower, if participation in routine chlamydia screening of HIV-infected MSM in care is suboptimal. The importance of sexual risk behaviour should be emphasized here. Figure 5 shows the percentage decline in HIV and chlamydia incidence with 100% participation in the routine screening program and with 80% participation in one of the four sexual risk groups. The impact of chlamydia screening is smaller if participation is suboptimal in the group with the highest risk behaviour, compared to the impact of screening if participation is suboptimal in the group with the lowest risk behaviour.

## Discussion

In this modelling study, we showed that routine chlamydia screening among HIV-infected MSM in care can result in reductions in chlamydia and HIV incidence in the overall MSM population. The impact of chlamydia screening on HIV incidence is a result of the increased HIV infectivity and susceptibility in individuals infected with chlamydia. The impact of chlamydia screening specifically among HIV-infected MSM in care can be explained by the fact that chlamydia incidence among HIV-infected MSM in care is much higher than among other MSM, due to the high density of high risk MSM within the HIV-infected population. Therefore, reducing chlamydia transmission in this core group will result in health gains not only for themselves, but also for the rest of the MSM population by preventing new chlamydia and HIV infections.

It should be stressed that the impact of chlamydia on HIV transmission shown here depends on the assumption of increased HIV infectivity and susceptibility due to chlamydia infection. This impact declines as the increase in HIV infectivity and susceptibility becomes smaller (Figures 2a,b and 4c,d); and if HIV infectivity and susceptibility are not altered at all in those with chlamydia infection, then chlamydia has no effect on HIV transmission. On the other hand, the decline in HIV incidence that we found due to chlamydia screening of HIV-infected MSM in care is not very high, as is the case with other interventions, such as widespread use of ART [18-20], or as previous studies have suggested [5,6]. This is a result of two factors. First, we assumed that HIV infectivity and HIV susceptibility are increased due to chlamydia infection by a factor of two, at most. Other modelling studies for heterosexuals have assumed higher increases [5,19], but we found no studies with significant evidence of such high increases [7-11]. Second, the increase in HIV infectivity due to chlamydia is diminished in individuals with undetectable viral load [9,21]; these individuals comprise the majority of the target group, since most of the HIV-infected MSM in care receive ART and most of them have undetectable viral load [14].

The impact of STIs on the HIV epidemic among heterosexuals has been investigated in several modelling studies (see, for instance, [6,22-26]). For MSM populations, this is the first study that addresses this issue and examines the impact of chlamydia screening specifically among HIV-infected MSM registered at HIV treatment centres. This is a high risk population, contributing the most to HIV and chlamydia transmission. Moreover, screening HIV-infected MSM in care can be easily implemented during the regular visits of these men at HIV clinics.

Certain limitations of this study should be mentioned. A test for anogenital chlamydia, in practice, may be carried out in combination with a test for gonorrhoea. Therefore, gonorrhoea infections may also be detected and treated, resulting in extra reduction in HIV transmission, since gonorrhoea also increases HIV infectivity and susceptibility [7,11]. From this point, our model may have underestimated the impact of routine chlamydia screening, as it does not account for the indirect effect of combined screening for chlamydia and gonorrhoea. Furthermore, some individuals treated for gonorrhoea, may also receive treatment for chlamydia; in that case the prevalence of chlamydia is reduced and consequently the impact of chlamydia screening could be lower.

Studies among individuals with recently acquired HIV infection have found high prevalences of chlamydia, suggesting that co-transmission of HIV and chlamydia could be possible and even frequent [27-29]. However, there are no data to verify that the two infections were acquired via the same sexual contact or to inform parameters relating to co-transmission. Therefore transmission of HIV and of chlamydia was modelled separately, such that co-transmission of the two infections is not possible and hence its potential impact is not accounted for in the model. Moreover, we did not account for orogenital transmission of HIV and chlamydia due to the lack of data. To avoid bias, we consistently restricted all related parameters and data to anogenital transmission, as much as possible. Nevertheless, for some parameters, data on heterosexual transmission were used, since there were no data for transmission between MSM.

Earlier modelling studies have shown that a substantial proportion of new HIV infections can be attributed to individuals with acute HIV infection [30,31], due to the high level of HIV infectivity during this short stage. This means that the impact of chlamydia might be overestimated in this study, since the impact is reduced with higher HIV infectivity (Figure 2d). Due to the complexity of the model, acute HIV infection was not included. Moreover, we did not account for differences in the duration of asymptomatic chlamydia between HIV-infected and HIV-negative MSM, because there are hardly any data on asymptomatic infections. Finally, in our calculations, we assumed a moderately assortative pattern of sexual mixing [32,33]; the role of mixing on the spread of STIs has been investigated in previous modelling studies (see, for instance, [34-37]).

After the introduction of routine chlamydia screening at HIV treatment centres, HIV-infected MSM in care may be less likely to be tested opportunistically outside the routine screening program. This means that HIV-infected MSM in care may not seek STI testing after symptoms or known risk of exposure, awaiting for the arranged regular visit at the HIV treatment centre. Moreover, opportunistic STI screening is mostly implemented at STI clinics, where also safe sex counselling and partner notification are offered. This means that introduction of routine chlamydia screening at HIV treatment centres may result in a reduction in opportunistic testing for other STIs, a reduction in partner notification, and, consequently, in increases in transmission of HIV and chlamydia. From this point, it is important that HIV-infected MSM in care who test positive for chlamydia during routine screening will still be offered a full STI consultation including safe sex counselling and partner notification.

These results have important implications for the design of public health policy interventions. Measures that can reduce chlamydia prevalence, such as chlamydia screening among HIV-infected MSM in care, should be promoted because they may also contribute to reducing HIV transmission. However, they should be considered as an addition to (and not as a substitute of) other measures to control HIV. Furthermore, it is essential that in particular high risk MSM participate in screening programs. Finally, it should be stressed that although data from the Netherlands were used for population characteristics in our model, such as sexual risk behaviour, our results were robust to changes in these characteristics. Hence, our findings apply also to other countries with considerable HIV and STI transmission among MSM.

## Conclusions

In conclusion, this analysis shows that chlamydia infection could have a considerable contribution to the population spread of HIV among MSM. Routine chlamydia screening of HIV-infected MSM at HIV treatment centres has the potential to reduce HIV and chlamydia incidence not only in the screened population of HIV-infected MSM in care, but among all MSM. Chlamydia screening will be more effective in reducing HIV incidence with more frequent testing or with higher participation of high-risk MSM in the screening program.

## Abbreviations

ART: Antiretroviral therapy; HIV: Human immunodeficiency virus; IQR: Interquartile range; MSM: Men who have sex with men; STI: Sexually transmitted infection; UAI: Unprotected anal intercourse.

## Competing interests

SEG has received in the past funding for consultancy by Gilead. For the remaining authors no other competing interests are declared.

## Authors’ contributions

MABVDS, JSF, JMP contributed to the study set up. MX developed the model, designed and carried out the model analyses, drafted the paper. HJV, MFSVDL assisted in the estimation of model parameters from literature and data. JW contributed to the statistical analysis of the model results. AKL, HJV, MFSVDL, MABVDS contributed to the design of the model structure and of the model scenarios. HJDV, JMP, SEG, BR, JSF, MP, MGVV contributed to the interpretation of data and results for HIV treatment centres, STI clinics, epidemiology of HIV and chlamydia. All authors contributed to the interpretation of results and to the writing of the paper. All authors read and approved the final manuscript.

## Pre-publication history

The pre-publication history for this paper can be accessed here:

http://www.biomedcentral.com/1471-2334/13/436/prepub

## Supplementary Material

Additional file 1: Table S1 Parameters relating to HIV and chlamydia infection. **Table S2.** Parameters relating to the sexual risk groups. **Table S3.** Parameters relating to the sexual risk behaviour and opportunistic chlamydia screening. **Figure S1.** HIV and chlamydia prevalence within each sexual risk group. **Figure S2.** Chlamydia prevalence according to HIV-serostatus. **Figure S3.** The incidence of HIV and chlamydia after a reduction in the frequency of opportunistic chlamydia screening. **Figure S4.** The incidence of HIV and chlamydia in each sexual risk group, after a reduction in the frequency of opportunistic chlamydia screening. **Figure S5.** Percentage change in HIV incidence with routine chlamydia screening among HIV-infected MSM in care plotted against the uncertain parameters. Click here for file
