# Supplementary material for: Modelling the impact of chlamydia screening on the transmission of HIV among men who have sex with men
Source: BMC Infect Dis. 2013 Sep 18;13:436. doi: 10.1186/1471-2334-13-436 (PMC3851177; doi:10.1186/1471-2334-13-436)
Supplement: Additional file 1: Table S1 — Parameters relating to HIV and chlamydia infection. Table S2. Parameters relating to the sexual risk groups. Table S3. Parameters relating to the sexual risk behaviour and opportunistic chlamydia screening. Figure S1. HIV and chlamydia prevalence within each sexual risk group. Figure S2. Chlamydia prevalence according to HIV-serostatus. Figure S3. The incidence of HIV and chlamydia after a reduction in the frequency of opportunistic chlamydia screening. Figure S4. The incidence of HIV and chlamydia in each sexual risk group, after a reduction in the frequency of opportunistic chlamydia screening. Figure S5. Percentage change in HIV incidence with routine chlamydia screening among HIV-infected MSM in care plotted against the uncertain parameters. [file 1471-2334-13-436-S1.doc]

**Modelling the impact of chlamydia screening on the transmission of HIV among men who have sex with men**

M. Xiridou, H.J. Vriend, A.K. Lugnér, J. Wallinga, J.S. Fennema, J.M. Prins, S.E. Geerlings,

B. Rijnders, M. Prins, H.J. de Vries, M.J. Postma, M.G. van Veen, M.F. Schim van der Loeff, M.A.B. van der Sande.

**Additional file**

**A.1. The course of HIV infection.** In the model, men who have sex with men (MSM) are divided into three classes according to the status of HIV infection: not infected with HIV, HIV-infected not in care, and HIV-infected in care. HIV-infected MSM not in care are those who are unaware of their infection or who are aware of their infection but they are not registered at a specialised HIV treatment centre. HIV-infected MSM in care are those who have been tested positive and are registered at HIV treatment centres; they receive counselling and they initiate antiretroviral therapy (ART) according to their CD4 counts and the current guidelines. In the model it is assumed that for those on ART with undetectable viral load, HIV infectivity is reduced by a factor $f_{A}^{'}$, while for the rest of the HIV-infected men in care infectivity is the same as for HIV-infected not in care. Let $\beta_{H}$ denote the probability of transmission of HIV per act of unprotected anal intercourse (UAI) from HIV-infected men not in care and $b$ the percentage of HIV-infected MSM in care with undetectable viral load. Then the probability of transmission of HIV from HIV-infected MSM with undetectable viral load is $f_{A}^{'}\beta_{H}$and from HIV-infected MSM in care is $f_{A}\beta_{H}$, where $f_{A}=bf_{A}^{'}+1-b$. The rate *θ* of "entering" care depends on testing rates and the willingness of those tested to register at specialised HIV treatment centres. For those with HIV, the rate of flowing out of the population is higher than for those without HIV due to HIV-related causes; moreover, this rate is lower for those in care than for those not in care, due to the longer life expectancy for those on ART.

**A.2. The course of chlamydia infection.** According to the status of chlamydia infection, individuals are divided into three classes: susceptible to chlamydia, symptomatic chlamydia, and asymptomatic chlamydia. Those with symptoms are treated and recover soon thereafter (at a rate $\gamma$). Those without symptoms remain undetected until natural recovery (at a rate $\delta$), unless they are found by opportunistic or routine screening (see below). After recovery (whether symptomatic or asymptomatic), immunity wanes and individuals are again susceptible to infection.

**A.3. Screening or testing for asymptomatic chlamydia.** Currently there is no routine screening for asymptomatic chlamydia in the Netherlands. However, MSM without symptoms related to sexually transmitted infections (STI) are tested for chlamydia and other STIs at their own initiative, at STI clinics or general practitioners [1-3]. This opportunistic screening for chlamydia is included in the model with the rate $\sigma_{\text{ij}}$, that may differ according to HIV status and according to sexual risk behaviour ($i=0,1,2$for those not infected with HIV, HIV-infected not in care, HIV-infected in care, respectively; $j=1,2,3,4$for the four sexual risk groups: low, fairly high, very high, extremely high). Finally, an extra screening rate $\sigma_{2j}^{'}$ is included in the model for HIV-infected MSM in care, to describe a new routine screening program where HIV-infected MSM in care will be tested for chlamydia during their regular visits at HIV treatment centres. After screening, men found positive for asymptomatic chlamydia receive treatment and recover.

**A.4. The interaction between HIV and chlamydia.** It is assumed that the presence of chlamydia increases the susceptibility to HIV (for those not infected with HIV) by a factor *φ*_1_ or *φ*_2_, depending on whether they have symptomatic or asymptomatic chlamydia. Also the presence of a chlamydia infection increases the infectiousness of HIV by a factor $v_{1j}$ for HIV-infected MSM not in care and by a factor $v_{2j}$ for HIV-infected MSM in care. Both $v_{1j}$ and $v_{2j}$ are defined for $j=1$ for those with symptomatic chlamydia and for $j=2$for those with asymptomatic chlamydia; for the uniformity of notation, we introduced in the equations also the factors $v_{10}=v_{20}=1$ for HIV-infected men without chlamydia. Studies among individuals receiving ART have shown that HIV infectivity is elevated due to chlamydia (compared to those without chlamydia) only for those with detectable viral load; for HIV-infected individuals with undetectable viral load, HIV infectivity is the same for those with and those without chlamydia. Therefore, in the model the factor $v_{2j}$ for HIV-infected MSM in care is defined as $v_{2j}=b+(1-b)v_{1j}$, for $j=0,1,2$, where $b$ is the fraction of HIV-infected MSM in care with undetectable viral load. The other characteristics of each infection were assumed to be unaffected by the presence of a second infection: the duration of symptomatic or asymptomatic chlamydia and the proportion of symptomatic chlamydia are the same for those with and those without HIV; the extra death rates due to HIV infection are also the same, in the presence or absence of chlamydia.

**A.5. The model for HIV and chlamydia transmission.** Figure 1 in the main text shows a flow diagram of the model for the transmission of HIV and chlamydia among MSM. Sexually active MSM are divided into nine classes with respect to HIV and chlamydia infection: those without any infection (*X*_00_); those with HIV only (*X*_10_, HIV-infected not in care; *X*_20_ HIV-infected in care); those with chlamydia only (*X*_01_, symptomatic; *X*_02_ asymptomatic); and those with both HIV and chlamydia (*X*_11_, *X*_12_, HIV-infected not in care with symptomatic or asymptomatic chlamydia, respectively; *X*_21_, *X*_22_, HIV-infected in care with symptomatic or asymptomatic chlamydia, respectively). For the variables *X_ij_* , the first subscript denotes status of HIV infection (0 is uninfected, 1 is infected not in care, 2 is infected in care) and the second subscript denotes status of chlamydia infection (0 is susceptible, 1 is symptomatic, 2 is asymptomatic).

Transmission occurs via UAI between men; other forms of transmission are not accounted for in this model. The population is stratified into four sexual risk groups according to the number of partners men have: groups 1, 2, 3, and 4 denoting the groups with low, fairly high, very high, and extremely high risk behaviour. Therefore, each of the nine categories (according to status of HIV and chlamydia infection) is further subdivided into the four activity groups: *X_ij_*_1_, *X_ij_*_2_, *X_ij_*_3_, and *X_ij_*_4_, are the numbers of men with *i* HIV status and *j* chlamydia status in the activity groups 1, 2, 3, and 4, respectively. In the model we distinguish three types of sexual partners: steady partners, single-act casual partners (casual partners with whom MSM have only one sexual contact and that is UAI), and multiple-acts casual partners (casual partners with whom MSM have more than one sexual contact, of which at least one is UAI).

The model is described by the following differential equations:

The rates $\lambda_{\text{Hi}}$ of HIV transmission and $\lambda_{\text{Si}}$ of chlamydia transmission are explained below. $N_{0}$ is the size of the MSM population and $\zeta_{i}$ is the fraction of the MSM population in sexual risk group $i$ = 1, 2, 3, 4, shown in Additional file 1: Table S2. Definitions and values of the other parameters are given in Additional file 1: Tables S1-S3.

**A.6. Rate of HIV transmission.** The rate at which MSM in risk group *i* get infected with HIV is defined separately for infection from steady partners (), infection from single-act casual partners (), and infection from multiple-acts casual partners (), from the following equations:

,

,

,

.

In these equations, *β_H_* is the probability of transmission of HIV per act of UAI and $v_{\text{ij}}$ are the factors increasing this probability for those with chlamydia (see section A4). Also, $a_{i}$ is the number of steady partners per year, $m_{\text{ij}}$ is the probability that a man of risk group *i* will choose a man from risk group *j* as steady partner, $u_{\text{ij}}$is the number of acts of UAI per year per partner for steady partnerships between a man of activity group *i* and a man of group *j*. For casual partners the parameters are as follows: $a_{i}^{'}$ and $a_{i}^{''}$ are the numbers of single-act and multiple-acts casual partners, $m_{\text{ij}}^{'}$ and $m_{\text{ij}}^{''}$ are the parameters for mixing with single-act and multiple-acts casual partners, and $u_{\text{ij}}^{''}$ is the number of acts of UAI per year per partner for multiple-acts casual partnerships between a man of activity group *i* and a man of group *j*. Finally, $h_{1}$ is a factor reducing the frequency of UAI due to knowledge of HIV infection for HIV-infected MSM not in care and $h_{2}$ is the respective factor for HIV-infected MSM in care.

**A.7. Rate of chlamydia transmission.** The rate at which men in activity group *i* get infected with chlamydia is defined apart for infection from steady partners (), infection from single-act casual partners (), and infection from multiple-acts casual partners (), from the following equations:

,

,,

,

where *β_S_* is the probability of transmission of chlamydia per act of UAI.

**A.8. Mixing in sexual partnerships.** Mixing between the sexual risk groups is defined by the parameters $m_{\text{ij}}$, $m_{\text{ij}}^{'}$, and $m_{\text{ij}}^{''}$, where $m_{\text{ij}}$ is the probability that a man of risk group *i* will choose a man from risk group *j* as steady partner, while $m_{\text{ij}}^{'}$ and $m_{\text{ij}}^{''}$ are the respective probabilities for single-act and multiple-acts casual partners. The $m_{\text{ij}}$ are defined by the following equations:

where $\delta_{\text{ij}}$is the Kronecker delta, being equal to 1, if $i=j$, and equal to 0, if $i\neq j$. The parameter $\varepsilon$ determines the level of assortativeness in mixing. If $\varepsilon=1$, then mixing is purely assortative, which means that men have partnerships only with men from their own activity group. If $\varepsilon=0$, then mixing is purely proportionate, which means that men choose their partners according to the ‘availability’ of partners. The term denotes the fraction of available partners from each risk group: $a_{j}$is the number of partners per year for risk group *j* and $N_{j}$ is the size of group *j*. A value of $\varepsilon$ greater than zero and less than one, ensures that a fraction $\varepsilon$ of partnerships is assortative (between men of the same risk group) and the remaining $1-\varepsilon$ partnerships are formed according to proportionate mixing. Similarly, the mixing parameters for single-act and multiple-acts casual partners are defined by the equations:

where $\varepsilon^{'}$ and $\varepsilon^{''}$ are the levels of assortativeness in mixing with single-act and multiple-acts casual partners; $a_{j}^{'}$, $a_{j}^{''}$ are the numbers of single-act and multiple-acts casual partners per year.

**A.9. The four sexual risk groups.** Parameters relating to sexual behaviour were mostly obtained from data from the Amsterdam Cohort Study among MSM. This is an open prospective cohort of MSM living in the region of Amsterdam [4]. Participants visit the Public Health Services of Amsterdam every six months to complete a self-reported questionnaire on sexual behaviour and to get tested for HIV. In this study, we used data from 2010 from 422 participants. Men were asked to report the number of steady and casual partners they had and whether they had UAI with these partners. Three types of casual partners were distinguished in the original questionnaire of the Amsterdam Cohort: (1) one-night stand (‘‘someone you have met by chance and had sex with only once’’), (2) multiple-time casual partner (‘‘someone you have met by chance on several occasions and had sex with on these occasions’’) and (3) sex buddy (‘‘someone you intentionally contact on a regular basis to have sex with’’) [5]. From the one-night stand casual partners we selected only those with whom men reported that they had UAI with; these partners are named in our study "single-act casual partners". From the multiple-time casual partners and the sex buddies we selected only those with whom men reported that they had at least once UAI with; these partners are named in our study "multiple-acts casual partners". Casual partners with whom men had no UAI were not included in our estimates of the number of casual partners. From the data, we calculated the number of steady partners, the number of single-act casual partners, and the number of multiple-act casual partners per year. The group of men reporting no UAI with casual partners was defined as risk group 1, the subgroup of the population with the "lowest" sexual risk behaviour. The remaining men were divided into risk groups 2, 3, 4 according to the total number of single-act and multiple-acts casual partners, with group 4 being the group with the "highest" sexual risk behaviour. Since men of risk group 1 have no UAI with casual partners, the parameters for the mixing with single-act casual partners $m_{\text{ij}}^{'}$ and for mixing with multiple-acts casual partners $m_{\text{ij}}^{''}$ are defined finally only for $i$ and$j$ equal to 2, 3, or 4.

**A.10. Parameter estimates.** Parameters relating to the biological characteristics of HIV and chlamydia were obtained from the literature and are summarised in Additional file 1: Table S1. In 2011, 8319 (97.6%) of the 8523 MSM registered at HIV treatment centres had started ART; the percentage of patients with virological suppression to below 50 copies/ml at 12 months after start of ART was 80.0% (95% CI, 78.4-81.6%) [6]. Hence, the fraction of HIV-infected MSM in care with undetectable viral load was b = 0.976*0.8 = 0.78. No data were available to estimate the frequency of UAI within steady and casual partnerships. The most relevant data found were the following. In the Netherlands, Rutgers WPF carries out an extensive study on sexual behaviour of the whole population [7]. From the survey carried out in 2011 data from men who identified themselves as MSM were extracted. Individuals were asked to report the frequency of sexual contacts with their sexual partners, from which we calculated an average of 55 sexual acts per man per year (from the answers of 353 MSM). These acts may not all be acts of UAI and they may not reflect the frequency of sex per partner that a man has but rather the frequency of sex with all the partners a man has within a year [8]. A similar estimate of an average of 59 sex acts per year was obtained from the frequency of sex (all types of sex, with all partners) reported by MSM not infected with HIV in 2006-2007 in the Bangkok MSM Cohort Study [9]. Based on these estimates, we assumed that the frequency of UAI between steady partners is $u$ per year per partner, where $u$ is in the range between 15 and 45, while the frequency of UAI between multiple-acts casual partners is $u^{''}$ per year per partner, with $u^{''}$ in the range between 1 and 15. Due to the lack of data we made no further assumptions about variations in the frequency of UAI according to sexual risk group of the two partners involved, which means that $u_{\text{ij}}=u$ and , for all $i$ and $j$.

**A.11. Uncertainty analysis.** After calculating the numbers of HIV-infected MSM in care from the model and their Poisson likelihoods, we weighted each outcome with the likelihood and calculated the weighted average. This weighted average corresponds to a Bayesian posterior mean. As a likelihood-based alternative to this Bayesian approach, we selected the parameter sets with likelihood higher than 0.00054 (1/8 of the saturated likelihood 0.0043212). The other sets were excluded from further analyses. The results of our Bayesian and our likelihood-based approach were very similar, with the likelihood-based approach being more robust to Monte Carlo sampling. Therefore, we report only the results of the likelihood-based approach. As an extra control for the influence of the threshold for making the selection of sets with high likelihood, we repeated all analyses with broader selections (selecting the parameter sets with likelihood higher than 10^-5^ or higher than 10^-3^); in both cases, the results were robust. Further, to ensure that the results are independent of the initial drawing of parameter values, we repeated the whole analysis: 10,000 new sets of parameter values were drawn from the respective ranges and the sets with likelihood higher than 1/8 of the saturated likelihood were selected; the results were also in agreement with the results of the first group of 10,000 parameter sets.

**A.12. The original 10,000 outcomes and calibration of the model to data.** From the original 10,000 parameter sets, the number of HIV-infected MSM in care varied from 232 to 37,657. The maximum of the 10,000 likelihoods of these estimates was 0.0043208 for an estimate of 8,524 HIV-infected MSM in care. Selecting the parameter sets with likelihood higher than 0.00054 resulted in a sample of 149 parameter sets where the number of HIV-infected MSM in care was between 8,336 and 8,710 (compared to 8,523 from data [6]). Furthermore, we also confirmed that the prevalences with the selected parameter values agreed well with those from data. Specifically, the median HIV prevalence from the model was 4.26% (see also Results in main text); from data, the prevalence of HIV in the Netherlands in 2008 was estimated at 5.05% (95% credible interval, 3.19-8.28%) [43]. The median prevalence of chlamydia from the model was calculated at 2.7% among MSM. This agrees with a prevalence of 3% found among young MSM (less than 30 years old) in three large cities in the Netherlands in 2008 [45].

**A.13. Variation in chlamydia and HIV prevalence and the impact of chlamydia screening.** Figures A1 and A2 show results with the selected parameter sets, for the current situation in the Netherlands: the prevalence of HIV and the prevalence of chlamydia in each sexual risk group (Additional file 1: Figure S1); and the prevalence of chlamydia according to HIV-serostatus (Additional file 1: Figure S2). The actual incidence of HIV and chlamydia for the scenario with reduced frequency of opportunistic chlamydia screening is shown in Figures A3 and A4. Results from the uncertainty analysis are shown in Additional file 1: Figure S5 for the percentage change in HIV incidence due to routine chlamydia screening of HIV-infected MSM in care. Routine screening is carried out every twelve months (black points), every six months (red points) or every four months (blue points). An increasing or decreasing pattern in the decline in HIV incidence was observed only with the five parameters shown here.

**References**

1. Stolte I. Sexual behaviour. In: "Final report of the health monitor of Amsterdam, 2008". Editors: Dijkshoorm H, van Dijk T, Janssen A. GGD Amsterdam, Amsterdam, 2009, pages 121-125.
2. Heiligenberg M, Rijnders B, Schim van der Loeff M, de Vries H, van der Meijden W, Geerlings S, Fennema H, Prins M, Prins J. High prevalence of sexually transmitted infections in HIV-infected men during routing outpatient visits in the Netherlands. Sex. Transm. Dis. 2012; 39:8-15.
3. van Empelen P, van Berkel M, Roos E, Zuilhof W. Schorer monitor 2011.
4. de Wit J, van den Hoek J, Sandfort T, van Griensven G. Increase in unprotected anogenital intercourse among homosexual men. Amer J Publ Health 1993; 83:1451-1453.
5. van den Boom W, Stolte I, Sandfort T, Davidovich U. Serosorting and sexual risk behaviour according to differenent casual partnership types among MSM: the study of one-night stands and sex buddies. AIDS Care 2012; 24:167-173.
6. van Sighem A, Smit C, Gras L, Holman R, Stolte I, Prins M and de Wolf F. ‘Monitoring of Human Immunodeficiency Virus (HIV) infection in the Netherlands Report 2011’, HIV Monitoring Foundation, Amsterdam, The Netherlands, 2011.
7. Wijsen C, de Haas S. Sexual health in the Netherlands in 2011: creating a representative sample for a population study. Tijdschrift voor Seksuologie 2012; 36:83-86. In Dutch.
8. de Graaf H. Sexual behaviour and feelings in the Netherlands. Tijdschrift voor Seksuologie 2012; 36:87-97. In Dutch.
9. van Griensven F, Thienkrua W, Sukwicha W, *et al*. Sex frequency and sex planning among men who have sex with men in Bangkok, Thailand: implications for pre- and post-exposure prophylaxis against HIV infection. J Intern AIDS Society 2010; 13:13.
10. DeGruttola V, Seage III G, Mayer K and Horsburgh Jr C. Infectiousness of HIV between male homosexual partners, J. Clin. Epidemiol. 1989, 42: 849–856.
11. Vittinghoff E, Douglas J, Judson F, McKirnan D, MacQueen K and Buchbinder S. Per-contact risk of human immunodeficiency virus transmission between male sexual partners, Amer. J. Epidem. 1999, 150: 306–311.
12. Baggaley R, White R and Boily MC. HIV transmission risk through anal intercourse: systematic review, meta-analysis and implications for HIV prevention, Int. J. Epidem. 2010, 39: 1048–63.
13. Wawer M, Gray R, Sewankambo N, *et. al.* Rates of HIV-1 transmission per coital act, by stage of HIV-1 infection, in Rakai, Uganda, J. Inf. Dis. 2005, 191: 1403–9.
14. Hendriks J, Craib K, Veugelers P, van Druten H, Coutinho R, Schechter M and van Griensven F. Secular trends in the survival of HIV-infected homosexual men in Amsterdam and Vancouver estimated from a death-included CD4-staged Markov model, Intern. J. Epid. 2000, 29: 565–572.
15. Geskus R. Methods for estimating the AIDS incubation time distribution when date of seroconversion is censored, Stat. Medic. 2001, 20: 795–812.
16. Morgan D, Mahe C, Mayanja B, *et. al*. HIV-1 infection in rural Africa: is there a difference in median time to AIDS and survival compared with that in industrialized countries?, AIDS 2002, 16: 597–603.
17. Collaborative Group on AIDS Incubation and HIV Survival. Time from HIV-1 seroconversion to AIDS and death before widespread use of highly-active antiretroviral therapy: a collaborative re-analysis, Lancet 2000, 355: 1131–7.
18. Antiretroviral Therapy Cohort Collaboration. Life expectancy of individuals on combination antiretroviral therapy in high-income countries: a collaborative analysis of 14 cohort studies, Lancet 2008, 372: 293–299.
19. Cohen M, Chen Y, McCauley M, *et. al.* Prevention of HIV-1 infection with early antiretroviral therapy, N. Engl. J. Med. 2011, 365: 493–505.
20. Wilson D, Law M, Grulich A and Kaldor J. Relation between HIV viral load and infectiousness: a model-based analysis, Lancet 2008, 372: 314–320.
21. Carr A, Chuah J, Hudson J, French M, Hoy J, Law M, Sayer D, Emery S and Cooper D. A randomised, open-label comparison of three highly active antiretroviral therapy regimens including two nucleoside analogues and indinavir for previously untreated HIV-1 infection: the OzCombo1 study, AIDS 2000, 14: 1171–1180.
22. Gray R, Li X, Wawer M, *et. al.* Stochastic simulation of the impact of antiretroviral therapy and HIV vaccines on HIV transmission; Rakai, Uganda, AIDS 2003, 17: 1941–51.
23. Quinn T, Wawer M, Sewankambo N, *et. al.* Viral load and heterosexual transmission of human immunodeficiency virus type 1, New Engl. J. Med. 2000, 342: 921–929.
24. Cohen M, Gay C, Kashuba A, Blower S and Paxton L. Narrative review: antiretroviral therapy to prevent the sexual transmission of HIV-1, An. Intern. Med. 2007, 146: 591–601.
25. Bezemer D, de Wolf F, Boerlijst M, *et. al.* A resurgent HIV-1 epidemic among men who have sex with men in the era of potent antiretroviral therapy, AIDS 2008, 22: 1071–1077.
26. Gras L, van Sighem A, Smit C, Zaheri S, Schuitemaker H and de Wolf F. ‘Monitoring of Human Immunodeficiency Virus (HIV) infection in the Netherlands Report 2007’, HIV Monitoring Foundation, Amsterdam, The Netherlands, 2007.
27. Kretzschmar M, Welte R, van den Hoek A and Postma M. Comparative model-based analysis of screening programs for chlamydia trachomatis infections, Am. J. Epidem. 2001, 153: 90–101.
28. Regan D, Wilson D and Hocking J. Coverage is the key for effective screening of Chlamydia trachomatis in Australia, J. Infect. Diseas. 2008, 198: 349–58.
29. Turner K, Adams E, Gay N, Ghani A, Mercer C and Edmunds W. Developing a realistic sexual network model of chlamydia transmission in Britain. Theor. Biol. Med. Model. 2006, 3:3.
30. Althaus C, Turner K, Schmid B, Heijne J, Kretzschmar M and Low N. Transmission of Chlamydia trachomatis through sexual partnerships: a comparison between three individual-based models and empirical data, J. R. Soc. Interface 2012, 9: 136–146.
31. Farley T, Cohen D and Elkins W. Asymptomatic sexually transmitted diseases: the case of screening, Prevent. Med. 2003, 36: 502–9.
32. McKay L, Clery H, Carrick-Anderson K, Hollis S and Scott G. Genital Chlamydia trachomatis infection in a subgroup of young men in the UK, Lancet 2003, 361: 1792.
33. De Vries R, van Bergen J, de Jong-van den Berg L, Postma M and the PILOT CT Study Group. Systematic screening for Chlamydia trachomatis: estimating cost-effectiveness using dynamic modeling and Dutch data, Val. Health 2006, 9: 1–11.
34. Van den Brule A, Munk C, Winther J, Kr¨uger Kjaer S, Jørgensen H, Meijer C and Morré S. Prevalence and persistence of asymtpomatic Chlamydia trachomatis infections in urine specimens from Danish male military recruits, Intern. J. STD AIDS 2002, 13 (Suppl. 2): 19–22.
35. Golden M, Schillinger J, Markowitz L and Louis M. Duration of untreated genital infections with Chlamydia trachomatis, Sex. Transmit. Dis. 2000, 27: 329–337.
36. Bernstein K, Marcus J, Nieri G, Philip S and Klausner J. Rectal gonorrhea and chlamydiaa reinfection is associated with increased risk of HIV seroconversion, JAIDS 2010, 53: 537–43.
37. Rottingen J, Cameron D and Garnett G. A systematic review of the epidemiologic interactions between sexually transmitted diseases and HIV: how much really is known?, Sex. Transm. Dis. 2001, 28: 579–97.
38. Jin F, Prestage G, Imrie J, *et. al*. Anal sexually transmitted infections and risk of HIV infection in homosexual men, JAIDS 2010, 53: 144–149.
39. Sadiq S, Taylor S, Kaye S, *et. al.* The effects of antiretroviral therapy on HIV-1 RNA loads in seminal plasma in HIV-positive patients with and without urethritis, AIDS 2002, 16: 219–25.
40. Rotchford K, Strum A and Wilkinson D. Effect of coinfection with STDs and of STD treatment of HIV shedding in genital-tract secretions: systematic review and data synthesis, Sex. Transm. Dis. 2000, 27: 243–8.
41. Berman S and Cohen M. STD treatment: how can it improve HIV prevention in the south?, Sex. Transm. Dis. 2006, 33: 850–7.
42. Kelley C, Haaland R, Patel P, *et. al*. HIV-1 RNA rectal shedding is recued in men with low plasma HIV-1 RNA viral loads and is not enhanced by sexually transmitted bacterial infections in the rectum, J. Inf. Dis. 2011, 204: 761–7.
43. Conti S, Presanis A, van Veen M, Xiridou M, Donoghoe M, Rinder Stengaard A and De Angelis D. Modeling of the HIV infection epidemic in the Netherlands: a multi-parameter evidence synthesis approach, An. Appl. Stat. 2011, 5:2359-2384.
44. Heijman T, Geskus R, Davidovich U, Coutinho R, Prins M, Stolte I. Less decrease in risk behaviour from pre-HIV to post-HIV seroconversion among MSM in the combination antiretroviral therapy era compared with the pre-combination antiretroviral therapy era. AIDS 2012, 26:489-95.
45. Van den Broek I, Hoebe C, van Bergen J, *et. al*. Evaluation design of a systematic, selective, internet-based, Chlamydia screening implementation in the Netherlands, 2008-2010: implication of first results for the analysis. BMC Infectious Diseases 2010, 10:89.

**Additional file 1: Table S1.** Parameters relating to HIV and chlamydia infection.

| **Symbol** | **Definition** | | **Values** | **Source** |
| --- | --- | --- | --- | --- |
| *Course of HIV infection* | | | | |
|  | Probability of transmission of HIV per act of UAI | 0.003-0.009 | | [10-13] |
|  | Duration of HIV infection until AIDS, for HIV-infected MSM not in care, years | 10 | | [13-17] |
|  | Duration of HIV infection until AIDS, for HIV-infected MSM in care, years | 40 | | [18] |
|  | Percentage of MSM in care with undetectable viral load | 80% | | [6] |
|  | Factor reducing HIV transmissibility for those on ART | 0.05 | | [19-24] |
|  | Factor reducing HIV transmissibility for those in care |  | |  |
|  | Average time from HIV infection till registration at HIV treatment centres, years | 4-6 | | [25,26] |
| *Course of chlamydia infection:* | | | | |
|  | Probability of chlamydia transmission per act of UAI | 0.10-0.24 | | [27-30] |
|  | Proportion of symptomatic chlamydia | 40% | | [27,31-33] |
|  | Duration of symptomatic chlamydia, months | 1 | | [27,33] |
|  | Duration of asymptomatic chlamydia, months | 8 | | [34,35] |
| *Factor increasing susceptibility to HIV due to infection with chlamydia:* | | | | |
|  | MSM with symptomatic chlamydia | 1-2 | | [36-38] |
|  | MSM with asymptomatic chlamydia | 1-2 | | [36-38] |
| *Factors increasing transmissibility of HIV due to co-infection with chlamydia:* | | | | |
|  | HIV-infected, without chlamydia | 1 | |  |
|  | HIV-infected not in care, with symptomatic chlamydia | 1-2 | | [39-41] |
|  | HIV-infected not in care, with asymptomatic chlamydia | 1-2 | | [39-41] |
|  | HIV-infected in care, with chlamydia |  | | [39,42] |

MSM, men who have sex with men; UAI, unprotected anal intercourse; ART, antiretroviral therapy; "in care" refers to HIV-infected MSM who are registered at HIV treatment centres and "not in care" to HIV-infected MSM who are not registered at such centres.

**Additional file 1: Table S2.** Parameters relating to the four sexual risk groups^a^.

| **Parameter** | **Sexual risk group** | | | |
| --- | --- | --- | --- | --- |
|  | **1** | **2** | **3** | **4** |
| Fraction of population in respective risk group | 70% | 20% | 7% | 3% |
| Number of steady partners per year | 0.27 | 0.26 | 0.32 | 0.54 |
| Number of single-act casual^b^ partners per year | 0 | 1.36 | 4.52 | 20.6 |
| Number of multiple-acts casual^c^ partners per year | 0 | 1.36 | 3.48 | 13.0 |

^a^ Estimated from data from the Amsterdam Cohort Study among men who have sex with men.

^b^ Casual partners with whom they had only one act of unprotected anal intercourse.

^c^ Casual partners with whom they had more than one act of unprotected anal intercourse.

**Additional file 1: Table S3.** Parameters relating to sexual risk behaviour and opportunistic chlamydia screening.

| **Symbol** | **Definition** | **Values** | **Source** |
| --- | --- | --- | --- |
|  | Sexual lifespan, years | 55 | a |
| *N*_0_ | Number of MSM in the Netherlands | 238000 | [43] |
| *Level of assortativeness in mixing in sexual partnerships* | | | |
|  | with steady partners | 75% |  |
|  | with single-act casual partners | 60% |  |
|  | with multiple-acts casual partners | 60% |  |
| *Number of acts of UAI per year per partner*^b^ | | | |
|  | for steady partners | 15-45 |  |
|  | for multiple-acts casual partnerships | 1-15 |  |
| *Factor change in number of acts of UAI per year per partner ($u$,*$u^{''}$), *due to knowledge of HIV infection* | | | |
|  | for HIV-infected MSM not in care | 1 |  |
|  | for HIV-infected MSM in care | 0.85 | [44] |
| *Opportunistic screening: average interval (in years) within which MSM without chlamydia symptoms get tested for chlamydia* | | | |
|  | HIV-infected in care^c^:  Risk group 1  Risk groups 2, 3, 4  Not infected with HIV & HIV-infected not in care:  Risk group 1:  Risk groups 2, 3, 4: | 2.5-3.5  1-1.5  2.5-3.5  1.5-2.5 | [1]  [1] |

MSM, men who have sex with men; UAI, unprotected anal intercourse.

^a^ Model assumption, accounting for 55 years of sexual lifespan (ages 15-69 years).

^b^  Estimate based on data from Rutgers WPF Group, resulting in an average frequency of 55 sex acts per year, among MSM in 2009 (see details in section A10).

^c^  Estimated from data from a pilot study on routine STI screening among HIV-infected MSM visiting two HIV treatment centres (Academic Medical Centre in Amsterdam and Erasmus Medical Centre in Rotterdam), from October 2007 through June 2008 [2].

**Additional file 1:**
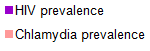

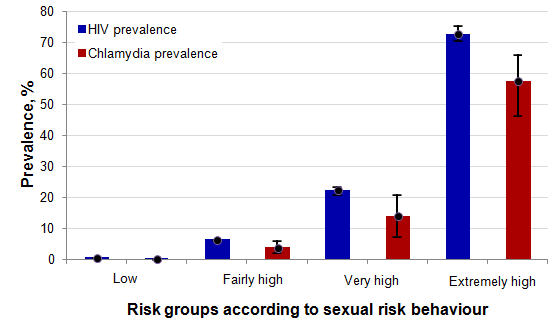
**Figure S1.** HIV prevalence (blue) and chlamydia prevalence (red) within each sexual risk group. The risk groups are ordered with increasing sexual risk behaviour according to the number of steady and casual partners (see details in Additional file 1: Table S2 and section A9). The height of the bars shows the median and the line segments show the interquartile range, calculated from the selected parameter values.

**Additional file 1:
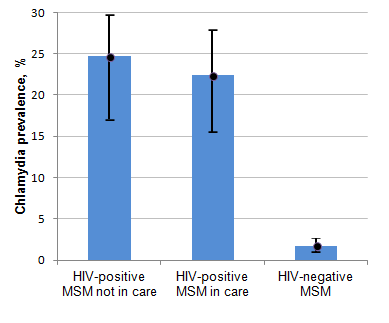
Figure S2.** The prevalence of chlamydia (%) among HIV-infected MSM not in care, among HIV-infected MSM in care, and among MSM not infected with HIV. The height of the bars shows the median and the line segments show the interquartile range, calculated from the selected parameter values.

HIV-infected MSM not in care

HIV-infected MSM in care

MSM not infected with HIV

**
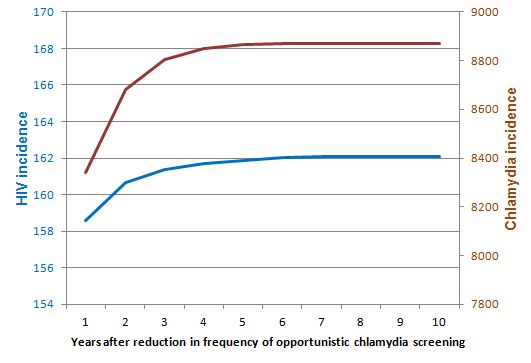
**

**Additional file 1: Figure S3.** The incidence of HIV (blue) and chlamydia (red) infection among MSM, after a reduction in the frequency of opportunistic chlamydia screening (the time interval between chlamydia tests as currently reported by MSM is increased in this scenario by 50%). The incidence is shown as number of new infections per year per 100,000 MSM; left vertical axis for HIV incidence, right vertical axis for chlamydia incidence..

**
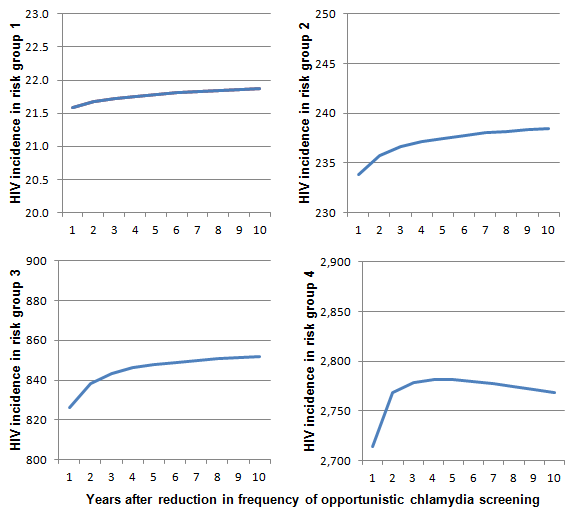
**

**Additional file 1: Figure S4.** The incidence of HIV infection among MSM, after a reduction in the frequency of opportunistic chlamydia screening (the time interval between chlamydia tests as currently reported by MSM is increased in this scenario by 50%). The horizontal axes show the number of years after the reduction in opportunistic screening. The incidence is shown as number of new HIV infections per year per 100,000 MSM within each sexual risk group: group 1 is low risk; group 2 is fairly high risk; group 3 is very high risk; and group 4 is extremely high risk.

**Additional file 1:**
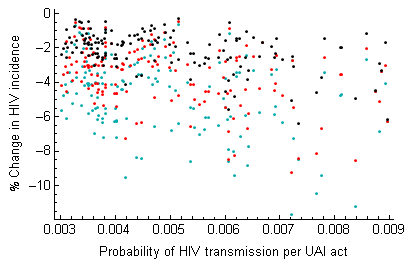

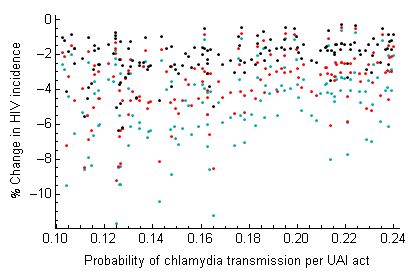

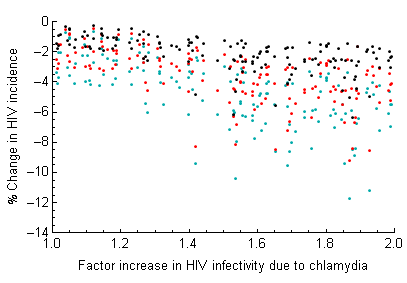

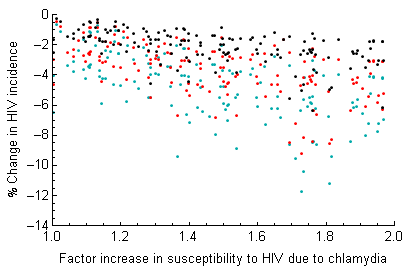

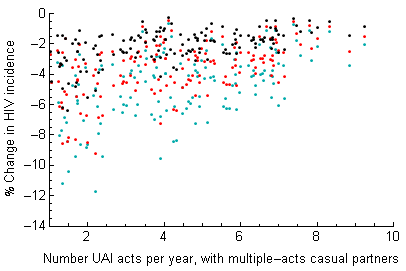
**Figure S5.** Percentage change in HIV incidence, ten years after the introduction of routine chlamydia screening among HIV-infected MSM in care. The routine screening is carried out every twelve months (black points), every six months (red points), or every four months (blue points). The percentage change is plotted against five of the uncertain parameters: the probability of HIV transmission per act of unprotected anal intercourse (UAI); the probability of chlamydia transmission per UAI act; the factor increase in HIV infectivity due to co-infection with chlamydia; the factor increase in susceptibility to HIV due to chlamydia; the number of UAI acts per year with multiple-acts casual partner. In each plot, one point corresponds to one of the selected parameter sets: the value of the uncertain parameter in this set can be viewed on the horizontal axis, while the percentage of new HIV infections attributed to chlamydia (as calculated from the model with the specific parameter value) is shown on the vertical axis.
